# Demographic analysis of fenpyroximate and thiacloprid exposed predatory mite *Amblyseius swirskii* (Acari: Phytoseiidae)

**DOI:** 10.1371/journal.pone.0206030

**Published:** 2018-11-15

**Authors:** Somayyeh Ghasemzadeh, Jawwad A. Qureshi

**Affiliations:** 1 Institute of Plant Protection, Chinese Academy of Agricultural Sciences, Haidian District, Beijing, P. R. China; 2 Department of Entomology and Nematology, Institute of Food and Agricultural Sciences, Indian River Research and Education Center, University of Florida, Fort Pierce, Florida, United States of America; Institut Sophia Agrobiotech, FRANCE

## Abstract

Knowledge of the impact of pesticides on predators is crucial for developing integrated pest management (IPM) programs. *Amblyseius swirskii* (Acari: Phytoseiidae) is a predatory mite used to control several species of pest including *Tetranychus urticae* (Acari: Tetranychidae) and arthropods. *T*. *urticae* is a major pest of multiple greenhouse-grown and field crops including apples in Iran. Lethal and sublethal effects of fenpyroximate and thiacloprid were investigated on *A*. *swirskii*, using these chemicals separately at recommended rates or in combination at reduced rates. Recommended tested rates of both pesticides negatively influenced the biological parameters of *A*. *swirskii* such as the net reproductive rate (*R*_*0*_) and the intrinsic rate of increase (*r*). However, the combined treatment of the two pesticides at their reduced rates was less hazardous to *A*. *swirskii*. Our findings indicate that the combined use of these chemicals may be compatible with IPM programs utilizing *A*. *swirskii* as biological control tool against phytophagous mites and other pests. However, semifield and field studies to investigate the effects of reduced rate treatments of fenpyroximate and thiacloprid alone and in combination on *T*. *urticae* and *A*. *swirskii* are required for developing IPM programs.

## Introduction

The two-spotted spider mite, *Tetranychus urticae* Koch (Acari: Tetranychidae), is a widespread, destructive and polyphagous pest of agricultural crops and ornamental plants worldwide [1–3]. Sever infestations could negatively influence quality and quantity of the fruit and more so for fresh production [4,5]. Predatory mites of the family Phytoseiidae are effective natural enemies of spider mites [6,7,8]. *Amblyseius swirskii* Athias-Henriot (Acari: Phytoseiidae) is a key predator of several major pests of agricultural crops and it is commercially available as a biological control agent. This species can develop and reproduce on a wide range of pests, including mites, thrips, whiteflies and moth eggs, as well as on various kinds of pollen [9–15].

Biological control and selective insecticides are important for developing an Integrated Pest Management (IPM) program and sustainable production system [16–18]. Use of biological control agents with selective pesticides could be an effective strategy to control pests and reduce the use of negative effects of synthetic pesticides. However, the use of conventional insecticides is common in several agro-ecosystems [19]. The sole reliance on synthetic pesticides could induce pest resistance, increase production cost and negatively impact environment, ecological services and human health [20–23]. Exposure to lethal or sublethal concentrations of pesticides may impact behavior, developmental rate, longevity, fecundity and sex ratio of the target pest species but also of non-target species, either harmful or beneficial [24–29]. Application of demographic analysis at the population level takes into account all the aforementioned effects that a toxicant might have on the target species [30,31,32]. This approach considers both lethal and sublethal effects and incorporate them into one endpoint, the intrinsic rate of natural increase [31,33,34], which is useful in detecting subtle, individual-level effects of contaminants that alter the growth of populations even at rates below the lethal concentration [35]. Few studies have been conducted to evaluate population growth rates of insect natural enemies in response to chemicals [36–40]. Therefore, knowledge of the toxicity of pesticides to beneficial organisms is important for effective pest management [29, 41].

Fenpyroximate and thiacloprid are widely used as acaricide and insecticide against many mites and insect pests of agricultural crops and ornamentals. The negative effects of fenpyroximate and thiacloprid on other beneficial mites and insects were reported [42,43,44]. However, evaluations against *A*. *swirskii* are limited. Only fenpyroximate was tested and fresh residues caused increased mortality of adults and larvae and decreased fecundity at high concentration [45].

Bearing this context in mind, we investigate lethal and sublethal effects of fenpyroximate and thiacloprid in independent treatments at the recommended rates, as well as in a combination at reduced rates, calculated with the non-linear programing on *A*. *swirskii* [46]. The non-linear programing is an analytical approach which helps identify and optimize limited production resources, under restricting conditions to obtain the most feasible benefit [47]. The knowledge of the effects of fenpyroximate and thiacloprid on *A*. *swirskii* could be useful to IPM programs against *T*. *urticae* [29].

## Materials and methods

### Rearing of mites

The colony of *T*. *urticae* mites was initiated from individuals collected from apple orchards of Urmia (West Azerbaijan province of Iran, in August and September 2015) and reared on bean plants (*Phaseoulus vulgaris* L. var. Talash) (Fabales: Fabaceae). No specific written permissions were required for field collections of mites because the owner had given us verbal permission to work in his orchard and make the collections. No permit or specific permission was required, because this study did not involve endangered or protected species. *Amblyseius swirskii* was provided by Koppert Biological Systems (Berkel en Rodenrijs, The Netherlands) and was reared on *T*. *urticae* on leaf disks of bean plants. The leaves were placed upside down on a wet sponge with a layer of cotton on the top in plastic trays with water (23×13 cm) and held in a climatic chamber at 25±1°C, 70±5% RH and a photoperiod of 16:8 (L:D) h. All experiments were conducted under these laboratory conditions.

### Pesticides

Fenpyroximate, commercial formulation Ortus 5% suspension concentrate, was provided by AGROXIR, Iran. Foliar sprays of fenpyroximate are used to control both immature and adult stages of mites [48]. Fenpyroximate is a mitochondrial electron transport inhibitor (METI) and affects target species by contact and ingestion. This pesticide is registered for the control of the European red mite, *Panonychus ulmi* Koch (Acari: Tetranychidae), and the two-spotted mite (*T*. *urticae*) on pome fruits, citrus, grapes and hops in several countries. Thiacloprid, commercial formulation Calypso 48% suspension concentrate, was provided by Bayer Crop Science, Germany. This chemical disrupts the nervous system of the target organism by inhibiting nicotinic acetylcholine receptors. Thiacloprid is used in the agricultural crops such as cotton and pome fruits to control a variety of sucking insects. Aphids and whiteflies are the primary target in cotton and psyllid, codling moth and plum Curculio in pome fruits [49].

### Lethal effect on adults

The acute toxicity and LC_50_ determinations were made on adult mites. In contact bioassay tests, commercial formulation of fenpyroximate and thiacloprid was applied at concentration of 0, 6, 11, 18, 30 and 50 mg a.i. liter^-1^ and 0, 6, 9, 13, 20 and 30 mg a.i. liter^-1^, respectively. Each concentration was replicated five times, with 10 individuals per replicate. Required solutions were prepared in distilled water. The concentrations of the pesticides were chosen based on the maximum field recommended concentration (MFRC) of these commercial compounds in Iran. The bean leaf discs (Ø 2.5 cm) were dipped in the tested pesticides’ solution for 10 s and allowed to dry for about three h under laboratory condition. The control leaf discs were dipped in distilled water. Mortality was recorded after 24 h of exposure. Mites were considered dead if they did not move when prodded with a soft paint brush.

### Sublethal effects on the progeny

The sublethal effects of fenpyroximate and thiacloprid were evaluated on the fecundity, survival and development of the progeny of the treated *A*. *swirskii*. The concentrations of the pesticides were prepared based on the MFRC in Iran. Fenpyroximate and thiacloprid in the independent treatments were used at 50 mg a.i. liter^-1^ and 30 mg a.i. liter^-1^, respectively, and in combination treatment at reduced rates of 17.2 mg a.i. liter^-1^ and 0.8 mg a.i. liter^-1^, respectively. These rates were determined using the non-linear programming framework for bean plants to estimate the optimum rates of two pesticides in the mixture to cause mortality of more than 50% to *T*. *urticae* and less than 50% to *A*. *swirskii* [46].

Mites were tested on freshly-excised bean leaf discs treated and control placed upside down in 30-ml transparent plastic cups containing water agar mixture (10%). The bean leaf discs were dipped for 10 s in the solution of each treatment (fenpyroximate, thiacloprid, fenpyroximate + thiacloprid combined at reduced rates) and in distilled water for control and allowed to dry for 3 h [50]. Cohort of fifty 24-h old females of *A*. *swirskii* from untreated bean plants was placed on leaf discs of each treatment and control. After 24 h, forty surviving females from each treatment and control were moved to untreated bean leaf discs at one per disc. Forty females were selected due to mortality of few individuals in some treatments and to use equal numbers across treatments. After 24 h, the eggs laid by each female from each of the experimental arena were placed in new arena at one egg per disc per female. The cohort of 0–24 h old eggs from each female was monitored through the development of nymphs and adults. Developing nymphs were provided with an abundant supply of *T*. *urticae* as prey. Ten adults of *T*. *urticae* were more than enough for the predator. The daily maximum food intake of one predatory mite was four adult spider mites [SG, personal observations]. Experimental arenas were checked daily to record survival and developmental time of the different life stages. The leaves were replaced every three days if it was necessary. Each newly emerged female from the four treatments (15–23 in each treatment) were coupled with an untreated male for mating under the conditions described above. Survival and fecundity was recorded until the death of the last individual.

### Statistical analysis

Mortality curves were estimated by probit analysis [51]. Raw data on the survival, longevity, and daily fecundity of individual females were analyzed using a life-stage specific and TWOSEX life table using both sexes in computer program MSChart [52–54]. The means and standard errors of the population parameters were estimated by a Bootstrapping procedure with 10,000 replicates [55–57]. The bootstrap method generates a normal distribution. Bootstrapping uses random resampling, otherwise a small number of replicates will generate variable means and large standard errors. Some data were analyzed using one-way analysis of variance (ANOVA) at *P* = 0.05. Differences between means were compared with the Tukey-Kramer (*P* = 0.05) procedure [58].

Following Chi and Liu [52], the age-stage specific survival rate (s_xj_), where x is age and j is the stage; the age-specific survival rate (l_x_); the age-specific fecundity (m_x_); the net reproductive rate (R_0_); the intrinsic rate of increase (r); the finite rate of increase (λ); the mean generation time (T) and the doubling time (DT) were calculated as follows:

$$l_{x}=\sum_{j=1}^{\beta }s_{xj}$$

$$m_{x}=\frac{\sum_{j=1}^{\beta }s_{xj}f_{xj}}{\sum_{j=1}^{\beta }s_{xj}}$$

Where β is the number of stages and *f*_*xj*_ is age-stage specific fecundity (where x = age and j = stage)

$$R_{0}=\sum_{x=0}^{\infty }l_{x}\;m_{x}$$

$$\sum_{x=0}^{\infty }e^{-r(x+1)}\;l_{x}\;m_{x}=1$$

$$\lambda =e^{r}$$

$$T=\frac{\text{ln}R_{0}}{r}$$

$$DT=\frac{\text{ln}2}{r}$$

## Results

### Lethal effect on adults

**Fig 1:**
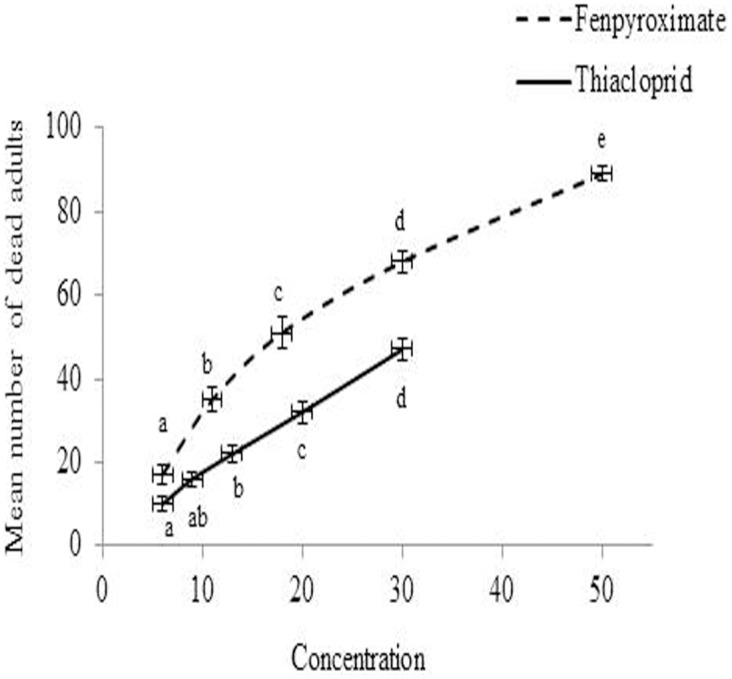
Mean (± SE) number of *Amblyseius swirskii* adults killed by residual concentrations of 6, 11, 18, 30 and 50 mg a.i. liter^-1^ for fenpyroximate and 6, 9, 13, 20 and 30 mg a.i. liter^-1^ for thiacloprid. A one-way ANOVA and Tukey’s mean separation test (*P* = 0.05) was performed to compare adult mortality among concentrations. Letters beside data points represent differences among concentrations of each pesticide.

The median lethal concentration (LC_50_) values for *A*. *swirskii* adults exposed to fenpyroximate and thiacloprid, were 16.67 mg a.i. liter^-1^ and 35.21 mg a.i. liter^-1^, respectively (Table 1). Mortality was significant in fenpyroximate (F = 104.66; df = 4, 20; *P* < 0.0001) and thiacloprid (F = 46.04; df = 4, 20; *P* < 0.0001) treatments averaging 89% and 47% after 24 h exposure at highest concentrations, respectively (Fig 1). No mortality was recorded in the control group.

**Table 1:** Median lethal concentration (LC_50_) estimated using probit analysis for adult female *Amblyseius swirskii* exposed to fenpyroximate and thiacloprid for 24 h.

Pesticide	95% Confidence limits	*χ*^2^	*Df*	LC(50)	Slope ± SE
Fenpyroximate	14.69–18.89	1.77	3	16.67	2.25 ± 0.20
Thiacloprid	27.49–53.58	0.36	3	35.212	1.70 ± 0.26

### Sublethal effects on the progeny

Overall biological parameters of the developing female and male progeny of females exposed to sublethal concentrations of fenpyroximate and thiacloprid were significantly affected compared with the control (Table 2). Duration of egg and larval stage of female and male from fenpyroximate alone treatment was significantly prolonged compared to those from control and treatments of thiacloprid alone and combination (*P* < 0.0001). However, there was no difference between treatments of fenpyroximate alone and thiacloprid alone for egg stage of female. Thiacloprid alone also prolonged the egg duration of female compared to control. Development time of each sex was significantly prolonged by each of the three treatments compared with control except combination against control for male (*P* < 0.0001). Female and male progeny of the females treated with fenpyroximate alone took significantly longer time to develop followed by those in thiacloprid alone, combination and control. Longevity of both sexes compared with control was significantly reduced in the treatment of fenpyroximate alone followed by thiacloprid alone and combination (*P* < 0.0001).

**Table 2:** Mean (±SE) developmental time, longevity and total life span (days) of offspring from females of *Amblyseius swirskii* from control or treatments of recommended rates of fenpyroximate and thiacloprid alone or in combination at reduced rates.

Sex/stage	Treatments					
	Fenpyroximate	Thiacloprid	Fenpyroximate+ Thiacloprid	Control	F (df,n)*	*P*
**Female**						
Egg duration	2.20 ± 0.13a	2.08 ± 0.15a	1.71 ± 0.13b	1.50 ± 0.14b	225.70	<0.0001
Larva duration	1.80 ± 0.25a	1.25 ± 0.13b	1.14 ± 0.10b	1.00 ± 0.00c	174.20	<0.0001
Protonymph	2.10 ± 0.18a	1.75 ± 0.13b	2.00 ± 0.15ab	2.00 ± 0.15ab	27.90	<0.0001
Deutonymph	2.10 ± 0.10a	2.00 ± 0.12a	2.14 ± 0.10a	2.00 ± 0.15a	7.20	0.0003
Developmental time	8.20 ± 0.29a	7.08 ± 0.19b	7.00 ± 0.23b	6.50 ± 0.23c	256.50	<0.0001
Longevity	15.80 ± 0.39d	20.67 ± 0.31c	21.64 ± 0.37b	23.50 ± 0.29a	2633.00	<0.0001
Total life span	24.00 ± 0.47d	27.75 ± 0.37c	28.64 ± 0.34b	30.00 ± 0.23a	1179.00	<0.0001
**Male**						
Egg duration	2.25 ± 0.16a	1.89 ± 0.11b	1.70 ± 0.15b	1.60 ± 0.16b	83.90	<0.0001
Larva duration	2.00 ± 0.27a	1.33 ± 0.17b	1.20 ± 0.13b	1.30 ± 0.15b	50.00	<0.0001
Protonymph	2.00 ± 0.27ab	1.78 ± 0.15b	2.00 ± 0.00a	2.00 ± 0.00a	27.00	<0.0001
Deutonymph	2.12 ± 0.12a	2.00 ± 0.00a	2.00 ± 0.15a	1.60 ± 0.22b	6.14	<0.0011
Developmental time	8.38 ± 0.32a	7.00 ± 0.00b	6.90 ± 0.28bc	6.50 ± 0.40c	157.40	<0.0001
Longevity	12.50 ± 0.33d	19.33 ± 0.29c	20.90 ± 0.55b	22.50 ± 0.54a	2111.00	<0.0001
Total life span	20.88 ± 0.35d	26.33 ± 0.29c	27.80 ± 0.39b	29.00 ± 0.33a	2685.00	<0.0001

Means followed by the same letter in the same row are not significantly different (Tukey-Kramer, *P* = 0.05)

*F: female (3,75), male (3,56)

Treatment of thiacloprid alone resulted in significantly prolonged pre-oviposition time compared with control (*P* < 0.0001, Table 3). Oviposition time was significantly reduced compared with control in all three treatments most with fenpyroximate alone followed by thiacloprid alone and combination (*P* < 0.0001, Table 3). Similar effect was observed for post-oviposition period and fecundity (*P* < 0.0001).

**Table 3:** Mean (±SE) reproductive period and total fecundity of offspring from females of *Amblyseius swirskii* from control or treatments of recommended rates of fenpyroximate and thiacloprid alone or in combination at reduced rates.

Parameters	Treatments					
	Fenpyroximate	Thiacloprid	Fenpyroximate+ Thiacloprid	Control	F (df,n)*	*P*
Pre-oviposition (day)	3.30 ± 0.15ab	3.50 ± 0.15a	3.21 ± 0.11b	3.14 ± 0.10b	381.05	<0.0001
Oviposition (day)	6.70 ± 0.26d	10.25 ± 0.25c	11.94 ± 0.30b	13.86 ± 0.25a	2199.64	<0.0001
Post-oviposition (day)	3.70 ± 0.14d	4.77 ± 0.12c	5.44 ± 0.13b	6.37 ± 0.12a	459.05	<0.0001
Total fecundity (no. eggs)	6.70 ± 0.25d	10.42 ± 0.30c	12.36 ± 0.35b	14.79 ± 0.40a	1562.25	<0.0001

Means followed by the same letter in the same row are not significantly different (Tukey-Kramer, *P* = 0.05)

*F (3,75)

**Fig 2:**
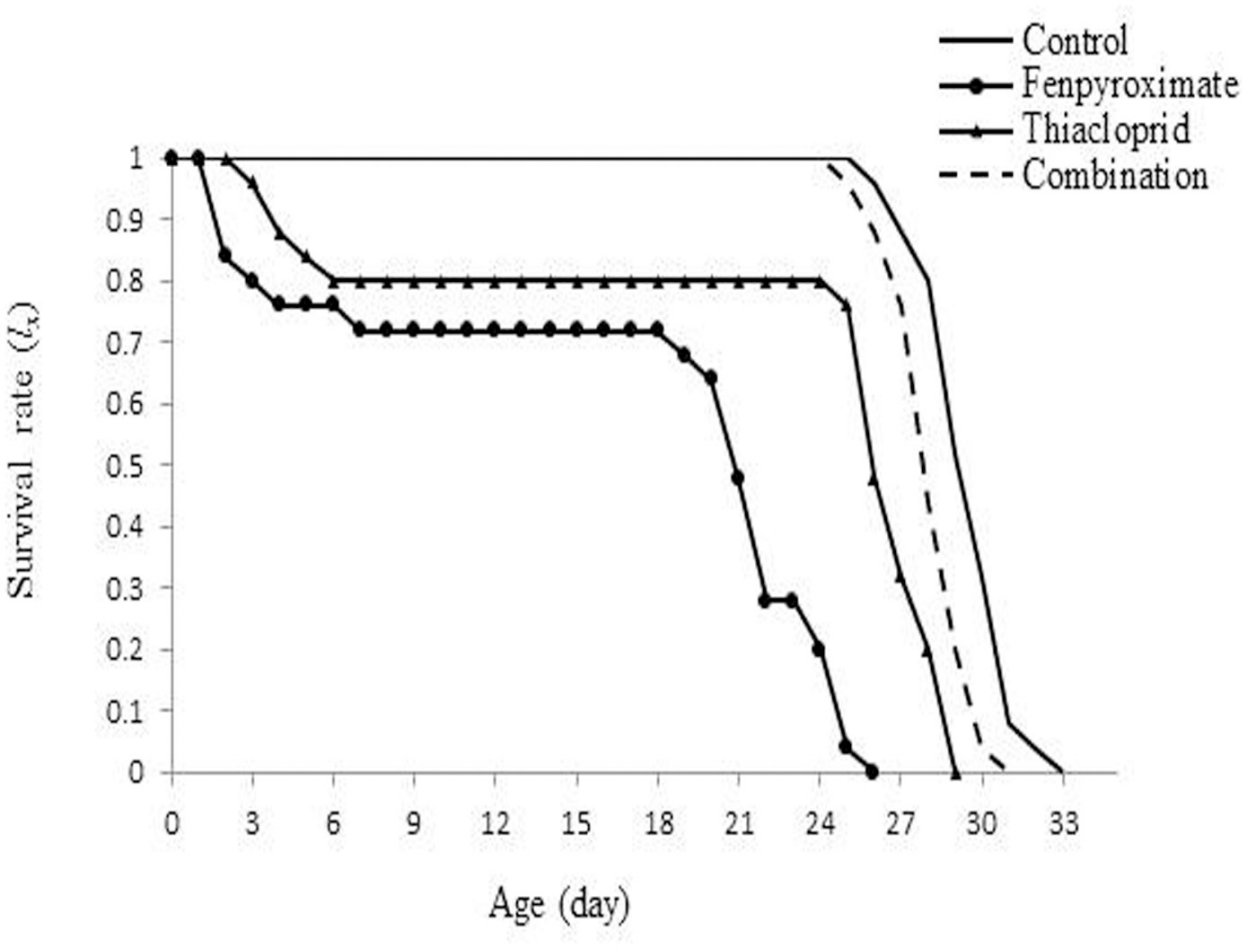
Age-specific survival (*l*_*x*_) of the population of *Amblyseius swirskii* females from control or treatments of recommended rates of fenpyroximate and thiacloprid alone or in combination at reduced rates. *l*_*x*_ represents the probability that an egg will survive to age x, and the curve of the age-specific survival rate is a simplified form of the curves of age-stage survival rate, regardless of developmental stage.

**Fig 3:**
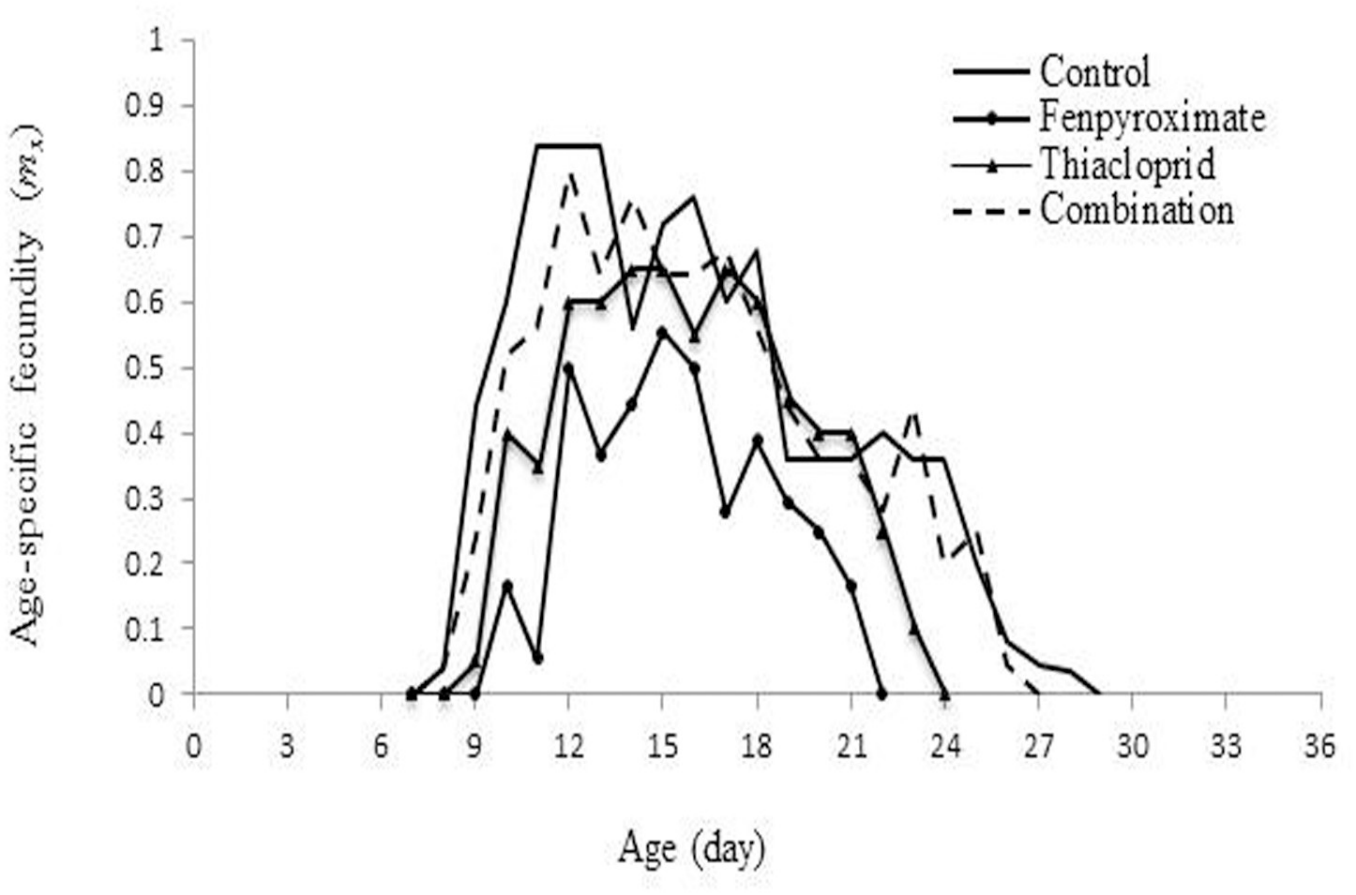
Age-specific fecundity (*m*_*x*_) of the population from *Amblyseius swirskii* females from control or treatments of recommended rates of fenpyroximate and thiacloprid alone or in combination at reduced rates.

Data on the age-specific survival rate (*l*_*x*_) and age-specific fecundity in all treatments are provided in Figs 2 and 3. Total life span averaged 30 days for the untreated females and 24 days, 27.75 days and 28.64 days for the females treated with fenpyroximate alone, thiacloprid alone and the combination at reduced rates, respectively. There was 2.5% mortality in the immature stages in the combination treatment, with 97.5% chance of reaching adulthood compared with control. In contrast, the mites treated with recommended concentrations of two pesticides showed highest mortality in immature stages, with 85 and 92.5% chances of reaching adulthood for fenpyroximate and thiacloprid, respectively.

**Fig 4:**
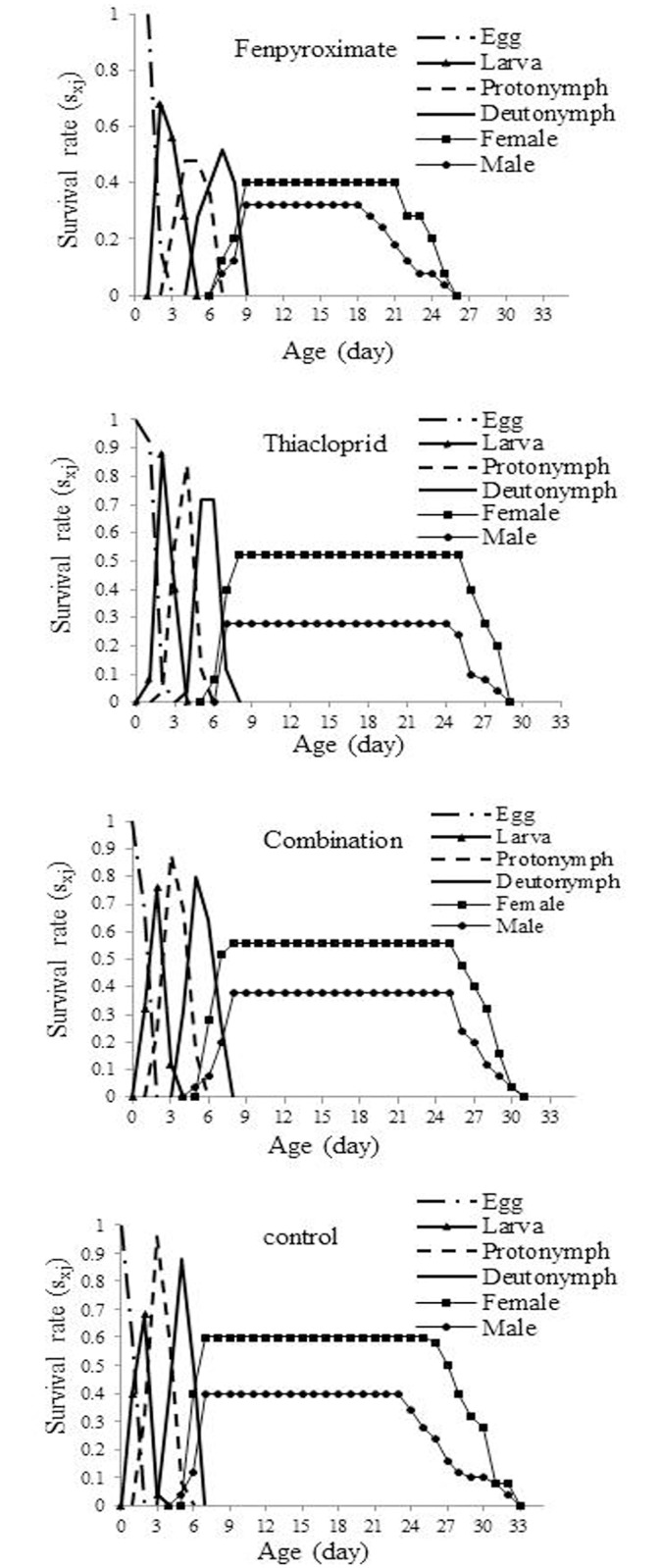
Age-stage specific survival rate (*s*_*xj*_) of the population from *Amblyseius swirskii* females from control or treatments of recommended rates of fenpyroximate and thiacloprid alone or in combination at reduced rates.

A maximum *mx* of 0.84 eggs/female/day was observed on day 11–13 of the untreated mites. For fenpyroximate alone, thiacloprid alone and combination treatment *mx* was approximately 0.55, 0.65 and 0.76 eggs/female/day, respectively, at 15, 14 and 14 day of the life span, respectively (Fig 3). Compared to the control, the fenpyroximate and thiacloprid treatments increased the duration of both egg and larval stages, and the pre-oviposition period. Male adults emerged earlier than females. The highest female survival rate was observed in the combination treatment and on average 56% of the eggs developed to the adult stage (Fig 4).

There was significant negative effect of the recommended rates of fenpyroximate and thiacloprid applied alone on the population parameters including intrinsic rate of increase (*r*), the finite rate of increase (*λ*), the net reproductive rate (*R*_*0*_), the gross reproductive rates (*GRR*), the mean generation time (*T*) and the doubling time (*DT*) (*P* < 0.0001, Table 4). However the effect of the combination treatment containing reduced rates of both pesticides compared to control were not statistically significant except for increase in *DT*.

**Table 4:** Mean (±SE) population parameters of the females of *Amblyseius swirskii* from control or treatments of recommended rates of fenpyroximate and thiacloprid alone or in combination at reduced rates.

Population parameters	Treatments					
	Fenpyroximate	Thiacloprid	Fenpyroximate+ Thiacloprid	Control	F (df,n)*	*P*
Intrinsic rate of increase, r (day^-1^)	0.06 ± 0.016c	0.09 ± 0.01b	0.12 ± 0.01ab	0.13 ± 0.01a	266.77	<0.0001
Finite rate of increase, λ (day^-1^)	1.06 ± 0.017c	1.10 ± 0.01b	1.13 ± 0.01ab	1.14 ± 0.01a	267.26	<0.0001
Net reproductive rate, R_0_ (offspring)	2.68 ± 0.66c	5.00 ± 1.04b	6.92 ± 1.24ab	8.28 ± 1.48a	233.27	<0.0001
Gross reproductive rate, GRR (offspring)	3.83 ± 0.79c	5.95 ± 1.14b	7.22 ± 1.26ab	8.62 ± 1.50a	112.67	<0.0001
Mean generation time, T (day)	16.31 ± 0.60a	16.16 ± 0.29a	16.11 ± 0.29ab	15.95 ± 0.26b	86.66	<0.0001
Doubling time, DT (day)	11.46 ± 0.15a	6.96 ± 0.26b	5.77 ± 0.21c	5.23 ± 0.25d	146.54	<0.0001

Means followed by the same letter in the same row are not significantly different (Tukey-Kramer, *P* = 0.05)

* F (3,156)

## Discussion

Determination of the compatibility of pesticides with natural enemies of plant pests is crucial for developing effective IPM strategies [24,59,60]. Assessment of the toxic effects of pesticides on natural enemies by measuring mortality rate alone underestimate the residual effects of the pesticides [61,62,63]. Knowledge of the population level effects of the pesticides on beneficial organisms is needed for developing integrated and sustainable pest management programs. Fenpyroximate and thiacloprid and several others pesticides are often used in combination and therefore selection of proper treatments is important [24,64]. Our study is the first evaluation of the effects of recommended field rate of fenpyroximate and thiacloprid applied alone and combination of the reduced rates of both pesticides on the demographic parameters of *A*. *swirskii*. The recommended rates prolonged egg and larval development of *A*. *swirskii* and reduced total life span. The combination treatment also reduced total life span. These effects may result in a reduced population growth of this predator as observed with reduced net reproductive rate and intrinsic rate of increase particularly at recommended rates, which could be significant to reduce biocontrol of pest mites. Overall, fenpyroximate had the strongest negative effect on *A*. *swirskii* followed by thiacloprid, whereas effects were strongly reduced or absent in the combination treatment of the two pesticides at reduced rates. Similar effects were observed on the eggs and larval development of *A*. *swirskii* when exposed to LC_30_ concentrations of fenazaquin, another insecticide with a mode of action similar to fenpyroximate [27]. The low-concentration strategy when effective against pests and compatible with biological control agents could be useful within an IPM program and to reduce selection pressure and the development of resistance [28,65]. There were no adverse effects of sublethal concentrations of spirodiclofen on developmental time, longevity and total life span of both sexes of *A*. *swirskii* [66]. Differences in phytoseiid species, populations, experimental method, pesticide mode of action, formulations and concentrations could be responsible for different results between studies. Fenpyroximate and fenazaquin functions as METI, thiacloprid acts on the nicotinic acetylcholine receptor (nAChR) [67,68,69] and spirodiclofen inhibits the acetyl-CoA carboxylase [70].

Reproductive variables of *A*. *swirskii* in all treatments except pre-oviposition period in the fenpyroximate treatment at recommended rate and combination treatment were reduced compared to the control. A negative impact of these and some other pesticides used at different rates on predatory mites including *A*. *swirskii* was also observed by other researchers. Lopez et al. [45] tested low lethal and sublethal concentrations of the proposed average field rate in bell peppers against *A*. *swirskii* under laboratory conditions and found that survival increased and fecundity decreased with the increase in concentration. Fecundity of *Phytoseius plumifer* Canestrini and Fanzago (Acari: Phytoseiidae) also decreased substantially with the increasing sublethal concentrations of fenpyroximate [71]. However, sublethal concentrations of spirodiclofen had no significant effects on the oviposition period and fecundity of *A*. *swirskii* [66]. Fecundity of *Phytoseiulus persimilis* Athias-Henroit (Acari: Phytoseiidae) was reduced when treated with sublethal concentrations of fenpyroximate [72]. Sublethal effects of fenpyroximate on *P*. *plumifer* and of fenazaquin on *A*. *swirskii* were also reported [27,71]. However, mortality of *Neoseiulus cucumeris* Oudemans (Acari: Phytoseiidae), *Typhlodromips montdorensis* Schicha (Acari: Phytoseiidae) and *A*. *swirskii* from direct applications and dry residues of thiacloprid and pymetrozine was similar to the control [73].

The age specific survival and fecundity of *A*. *swirskii* were reduced in the full rate treatments of fenpyroximate and thiacloprid but not much in the combination treatment at reduced rates. Exposure of *P*. *plumifer* to fenpyroximate and abamectin at the highest recommended field concentration produced similar effects [74]. Fecundity of acequinocyl exposed *Typhlodromus pyri* Scheuten (Acari: Phytoseiidae), was also reduced [75], however, fecundity and some other demographic parameters of *A*. *swirskii* exposed to spirodiclofen were not influenced suggesting that sublethal concentrations may not affect the population parameters of offspring from treated *A*. *swirskii* [66]. Due to the variability in the developmental rate among individuals, the survival curve of predatory mites treated with pesticides showed significant stage over-lapping in our study and others [26,27,66]. Some of the discrepancies in findings between studies could be due to use of different pesticide and the mite response to those.

Our results revealed significant differences in population growth and reproductive rates between the treated and untreated females of *A*. *swirskii*. The population parameters including the intrinsic rate of increase (*r*_*m*_), finite rate of increase (λ), net reproductive rate (R_0_) and gross reproductive rate (GRR) of the *A*. *swirskii* were reduced in the full rate treatments of fenpyroximate and thiacloprid compared with the mites in control but not in the combination treatment. A noticeable reduction of these population parameters was observed with fenpyroximate than thiacloprid. Similar effects of these and some other pesticides against *A*. *swirskii* and other species were also observed by other researchers. For example, adverse impacts of chlorantraniliprole, cyantraniliprole and lambda-cyhalothrin on the *r*_*m*_ of *Chrysoperla carnea* (Stephens) (Neuroptera: Chrysopidae) and *Trioxys pallidus* (Haliday) (Hymenoptera: Braconidae) [76]; and of lethal concentrations of spiromesifen and spirodiclofen on the *r*_*m*_ of *Neoseiulus californicus* McGregor (Acari: Phytoseiidae) [28]. However, frequent sprays of hexythiazox had no significant effect on *r*_*m*_ of *P*. *persimilis* for several generations [77]. Hamedi et al. [71] demonstrated that *P*. *plumifer* treated with LC_10_, LC_20_ and LC_30_ concentrations of fenpyroximate had significantly reduced λ than the untreated mites also observed in similar studies against *N*. *californicus* [78]. Our findings of reduced *R*_0_ and GRR of *A*. *swirskii* in the treatments of full rates were similar to other reports indicating that the lethal and sublethal concentration of acaricides significantly reduced the *R*_0_ of phytoseiids [26,27,28,79]. The full treatments of fenpyroximate and thiacloprid prolonged the mean generation time (*T*) of *A*. *swirskii* compared with control but not when combined at the reduced rates. Similar effect on *A*. *swirskii* was seen with the sublethal concentration of fenazaquin [27]. Noticeably prolonged doubling time (*DT*) of *A*. *swirskii* was observed from exposure to full rate treatments of fenpyroximate and thiacloprid than combination treatment of their reduced rates.

Fenpyroximate and thiacloprid at recommended rates compared to the control negatively influenced the biological parameters of *A*. *swirskii*. The combination of the reduced rates of both pesticides was less hazardous to *A*. *swirskii* and may be useful within an IPM program utilizing *A*. *swirskii* for biological control of phytophagous mites and other pests. However, semifield and field studies are needed to investigate the level of compatibility between the concentrations of fenpyroximate and thiacloprid and *A*. *swirskii* and to assess their efficiency in controlling *T*. *urticae*.
